# Brain networks involved in the influence of religion on empathy in male Vietnam War veterans

**DOI:** 10.1038/s41598-021-90481-3

**Published:** 2021-05-26

**Authors:** Irene Cristofori, Wanting Zhong, Shira Cohen-Zimerman, Joseph Bulbulia, Barry Gordon, Frank Krueger, Jordan Grafman

**Affiliations:** 1 Institute of Cognitive Sciences Marc Jeannerod CNRS, UMR 5229, 67 Boulevard Pinel, 69675 Bron, France; 2 University of Lyon, Etablissement 1, Villeurbanne, France; 3 Cognitive Neuroscience Laboratory, Brain Injury Research, Shirley Ryan AbilityLab, Chicago, IL USA; 4 Department of Physical Medicine and Rehabilitation, Northwestern University, Chicago, IL USA; 5 School of Psychology, Faculty of Science, Victoria University of Wellington, Wellington, New Zealand; 6 Max Plank Institute for the Science of Human History, Jena, Germany; 7 Department of Neurology, Johns Hopkins University School of Medicine, Baltimore, MD USA; 8 Department of Cognitive Science, Johns Hopkins University, Baltimore, MD USA; 9 School of Systems Biology, George Mason University, Fairfax, VA USA; 10 Department of Psychology, George Mason University, Fairfax, VA USA; 11 Department of Neurology, Feinberg School of Medicine, Chicago, IL USA; 12 Department of Psychiatry, Feinberg School of Medicine, Chicago, IL USA; 13 Department of Cognitive Neurology & Alzheimer’s Disease, Feinberg School of Medicine, Chicago, IL USA; 14 Department of Psychology, Northwestern University, Chicago, IL USA

**Keywords:** Emotion, Social behaviour, Social neuroscience

## Abstract

Humans all over the world believe in spirits and deities, yet how the brain supports religious cognition remains unclear. Drawing on a unique sample of patients with penetrating traumatic brain injuries (pTBI) and matched healthy controls (HCs) we investigate dependencies of religious cognition on neural networks that represent (1) others agents’ intentions (Theory of Mind, ToM) and (2) other agents’ feelings (Empathy). Extending previous observations that ToM networks are recruited during prayer, we find that people with vmPFC damage report higher scores on the personal relationship with God inventory even when they are not praying. This result offers evidence that it is the modulation of ToM networks that support beliefs in supernatural agents. With respect to empathetic processing, we observed that vmPFC and pSTS/TPJ lesions mediated by the strength of the personal relationship with God affect empathetic responses. We suggest that the neurological networks underpinning God representations amplify human empathetic responses. The cultural evolutionary study of religion has argued that supernatural beliefs evoke pro-social responses because people fear the wrath of Gods. Our findings imply greater attention should be paid to the mechanisms by which religious cognition may regulate empathetic responses to others.

## Introduction

Humans are a religious species. Why religions endure over evolutionary timescales remains a topic of enduring speculation^1^. One explanation links religious cognition to prosocial behavior^2,3^, since belief in divine punishments drives altruistic behaviors by fostering anti-social restraint [for a review see Ref.^4^]. A belief in moralistic, punitive and knowing Gods is also associated with impartial behavior towards geographically distant religious believers, thus helping expand the range of social cooperation^5^ and greater religious commitment leads to more altruistic behaviors^6,7^. Notably, however, the evidence about the mechanisms linking religious belief to anti-social restraint is mixed. While research utilizing self-report measures of prosociality has found positive relationships between religious beliefs and altruistic behaviors, experimental research using objective measures of prosociality (e.g., behavioral economic paradigms) has proved less conclusive^8^. In addition, priming religious words increased prosocial behavior^2^, but in some contexts, religious primes increased aggressive revenge^9,10^ and hostility toward outgroups^11^.

For this reason, it has been suggested that prosocial religious behavior may also drive empathetic responses^12^. In addition, despite the prevalence of religion all over the world, social affective neuroscience has yet to systematically investigate the neural basis of religious cognition^13,14^. Two possible explanations link religiosity to prosociality, one is fostering anti-social restraint, one is driving empathetic response. They are not mutually exclusive. In this study, we examine the hypothesis on empathy.

Here, we investigate the neural interplay between religious beliefs and empathy. Empathy is a multi-faceted construct that describes the capability to infer and understand and represent others’ feelings^15^. It is composed of an affective empathy component, which enables sharing and responding to another’s emotional state, and a cognitive empathy component, which allows one to take someone else’s perspective. The cognitive component is facilitated by Theory of Mind (ToM), which is the ability to attribute mental states to others different from oneself^16^. ToM is processed in a network of regions involving the temporoparietal junction (TPJ), the superior temporal sulcus (STS)^17–19^ and dorsolateral prefrontal cortex (dlPFC)^20,21^, with this latter region involved in the cognitive aspects related to TOM. It has been proposed that belief in God requires mentalizing abilities, since Gods are represented in a way similar to how the mental states of other humans are perceived^22–24^.

Many religions endorse prosocial and helping behavior^25^, and this orientation towards others is shared by the core components of empathy. Previous studies have identified a direct association between religiosity and helping behavior as well as empathy^26,27^. More recent theories support these early studies and view religious beliefs and behaviors as complex socio-cognitive phenomena that *require* higher social functioning abilities such as empathy and ToM^28,29^. In addition, functional neuroimaging evidence has identified that the ToM network, including the inferior and middle frontal gyrus, and the inferior temporal gyrus, is crucial when interpreting the actions of God^22^. Other brain regions, such as the ventromedial prefrontal cortex (vmPFC), have been specifically associated with empathy^30^ and prosocial behavior^31–33^. The vmPFC role in empathy and prosocial behavior is dependent on its more general role in encoding stimulus value^34–36^.

Despite a growing body of research investigating the brain bases of religious beliefs^13^, the neurological links between empathy and religious belief have yet to be systematically investigated. To study this relationship, we administered an abbreviated version of the God Image Inventory^37^ to a sample of male Vietnam combat veterans divided into those with focal penetrating Traumatic Brain Injury (pTBI, *n* = 109) and those with no injuries (*n* = 31). The God Image Inventory is a valid, standardized psychometric measure of the internalized relationship an individual has with God^38^. This cohort of Vietnam combat veterans with pTBI is unique given its large size, and its extensive neuropsychological and experimental dataset, including a pre-injury intelligence measure. First, in agreement with previous studies^38^ we hypothesized that lesions to the vmPFC would be associated with a strengthened internalized God image (i.e., quantified using the God Image Inventory). Second, we hypothesized that a strengthened internalized God image would positively correlate with lesion volume and empathy scores in the vmPFC group. Third, we tested two alternative hypotheses for the interplay between empathy and religion using mediation models. Some theories propose that religious beliefs are a cultural product promoted by existent human psychological tendencies, such as detecting the content of other minds (theory of mind) or enhancing one’s prosocial reputation within a group^39^ (Hypothesis 1). In contrast, other theories have suggested that religious beliefs can act as an engine to promote greater prosocial tendencies in humans^4,40^ (Hypothesis 2). Using lesion locations as independent variables, to test Hypothesis 1, we entered empathy as the mediator and internalized God image as the dependent variable. Alternatively, to test Hypothesis 2, we entered internalized God image as the mediator and empathy as the dependent variable.

Previous studies have shown that vmPFC lesions are associated with lower empathy^25,26^, however to which extent empathy is modulated by religious beliefs is still unknown. Since, in general, patients with vmPFC lesions report stronger religious beliefs but lower empathy, we wondered whether stronger religious beliefs could modulate empathy level, supporting Hypothesis 2.

## Results

### Descriptive statistic results

The following tables (Tables 1, 2) summarize demographic characteristics and neuropsychological tests among groups.

**Table 1:** Description of demographic characteristics and neuropsychological tests [mean ± SD], and t-test statistics for pTBI patients and HCs.

	pTBIs (n = 109)	HCs (n = 31)	Statistics
Age (years)	63.42 ± 3.02	63.06 ± 3.46	t(138) = 0.56, p = 0.574, Cohen’s *d* = 0.111
Education (years)	14.75 ± 2.25	15.13 ± 2.17	t(138) = − 0.82, p = 0.409, Cohen’s *d* = 0.172
Handedness	1.19 ± 0.44	1.26 ± 0.57	t(138) = − 0.67, p = 0.498, Cohen’s *d* = 0.137
Pre-injury AFQT	66.36 ± 22.80	70.76 ± 17.63	t(138) = − 0.82, p = 0.210, Cohen’s *d* = 0.215
Post-injury AFQT	57.80 ± 25.32	72.50 ± 19.42	t(138) = − 2.94, p = 0.004, Cohen’s *d* = 0.651
Verbal fluency	8.96 ± 3.49	10.41 ± 3.46	t(138) = − 1.98, p = 0.049, Cohen’s *d* = 0.417
Token test	98.12 ± 2.55	98.42 ± 1.87	t(138) = − 0.60, p = 0.549, Cohen’s *d* = 0.134
Beck Depression Inventory II	6.98 ± 7.26	10.00 ± 8.69	t(138) = − 1.95, p = 0.053, Cohen’s *d* = 0.377
Mississippi PTSD Scale	76.38 ± 20.72	82.27 ± 22.42	t(138) = − 1.95, p = 0.052, Cohen’s *d* = 0.272

The AFQT represents the Armed Forces Qualification Test—measure of intellectual ability; PTSD is used to represent Post-traumatic stress disorder.

pTBIs and HCs are used to represent penetrating traumatic brain injury and healthy controls.

**Table 2:** Description of demographic characteristics and neuropsychological tests [mean ± SD], and ANOVA test statistics for pTBI patients in lesion subgroups and HCs^a^ (^a^For HCs scores, see Table 1).

	vmPFC (n = 14)	dlPFC (n = 15)	pSTS/TPJ (n = 17)	Statistics
Age (years)	62.71 ± 2.74	63.29 ± 2.58	63.24 ± 1.67	F(3,73) = 0.12, p = 0.946, η_p_^2^ = 0.005
Education (years)	13.85 ± 2.31	14.43 ± 2.47	14.94 ± 1.91	F(3,73) = 1.25, p = 0.297, η_p_^2^ = 0.050
Handedness	1.07 ± 0.26	1.36 ± 0.49	1.18 ± 0.52	F(3,73) = 0.85, p = 0.469, η_p_^2^ = 0.034
Pre-injury AFQT	52.64 ± 32.51	68.18 ± 23.29	70.20 ± 17.90	F(3,73) = 1.84, p = 0.149, η_p_^2^ = 0.090
Post-injury AFQT	54.28 ± 33.26	71.76 ± 13.88	73.82 ± 17.58	F(3,73) = 2.36, p = 0.078, η_p_^2^ = 0.090
Verbal fluency	8.14 ± 2.41	8.36 ± 3.50	10.24 ± 3.46	F(3,73) = 1.75, p = 0.163, η_p_^2^ = 0.068
Token test	97.85 ± 2.87	98.36 ± 1.94	98.35 ± 2.39	F(3,73) = 0.20, p = 0.899, η_p_^2^ = 0.008
Beck Depression Inventory II	10.42 ± 10.44	4.36 ± 4.32	7.82 ± 6.47	F(3,73) = 2.02, p = 0.118, η_p_^2^ = 0.078
Mississippi PTSD Scale	83.92 ± 22.37	66.36 ± 14.89	77.59 ± 24.21	F(3,73) = 2.44, p = 0.071, η_p_^2^ = 0.093

*vmPFC* ventromedial prefrontal cortex, *dlPFC* dorsolateral prefrontal cortex, *pSTS/TPJ* posterior superior temporal sulcus/temporo-parietal junction.

### Group analysis

First, we performed two-sample independent t-tests to compare the scores on the God Image Inventory (religious belief) and Interpersonal Reactivity Index (Empathy) between pTBI and HC. Second, we performed one-way ANOVAs to compare God Image Inventory and Interpersonal Reactivity Index scores among the different lesion subgroups and HC. We complemented our use of frequentist statistical analyses by also examining results using a Bayesian approach.

The pTBI group (M = 0.068 ± 0.929) reported a higher God Image Inventory score compared to the HC group (M = − 0.333 ± 1.188) (t(139) = 1.994, p = 0.048, Cohen’s *d* = 0.245). The corresponding Bayesian two-sample t-tests on the same God Image score demonstrated substantial support for the alternative hypothesis (BF_10_ = 6.40, error % = 0.001).

The pTBI group (M = 83.20 ± 10.94) and the HC group (M = 79.38 ± 10.79) reported similar Interpersonal Reactivity Index scores (t(137) = 1.677, p = 0.096, Cohen’s *d* = 0.350). The corresponding Bayesian two-sample t-tests on the same Interpersonal Reactivity Index scores did not demonstrate support for the alternative hypothesis (BF10 = 0.75, error % = 1.053e−4).

**Figure 1:**
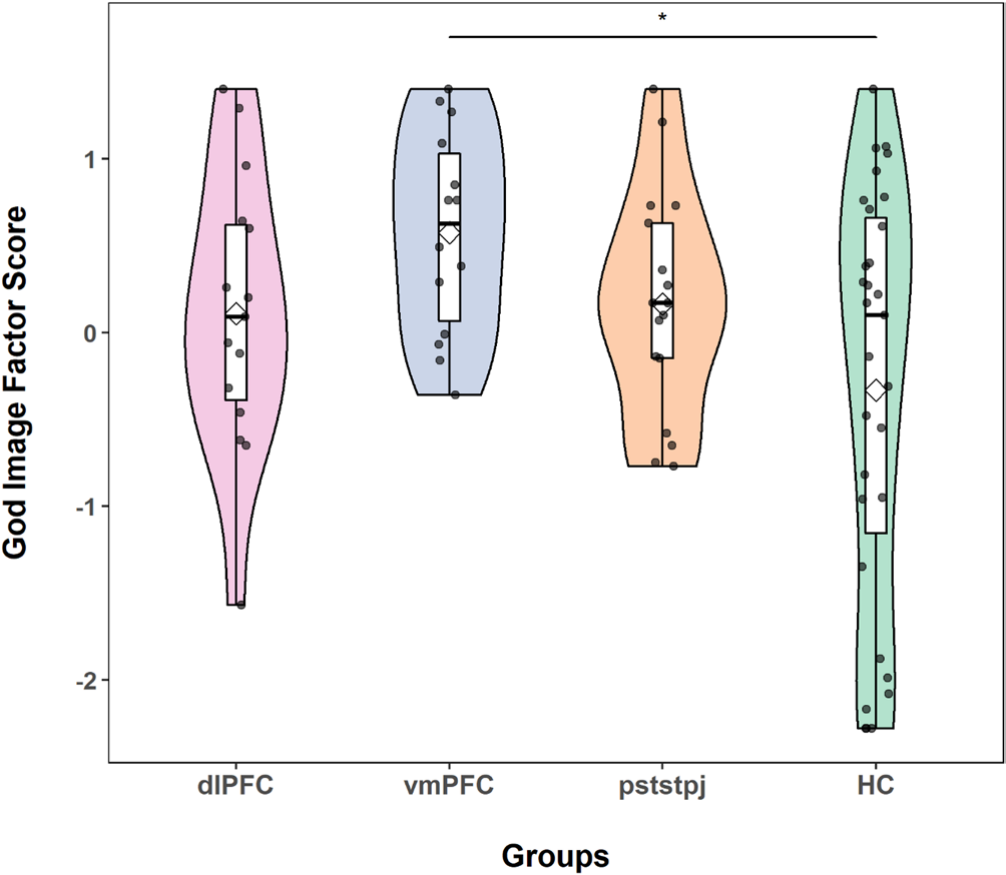
God Image Factor Score violin/boxplots for the different groups. Participants with vmPFC damage demonstrated higher scores on the God Image scale than HCs (p < 0.05). The boxes indicate the 75th percentile (upper horizontal line), the mean of each group (diamond), the median for each group (line), and the 25th percentile (lower horizontal line) of the distribution. Each dot indicates a participant. The upper whiskers indicate the maximum value of the variable located within a distance of 1.5 times the interquartile range above the 75th percentile. The lower whiskers indicate the corresponding distance to the 25th percentile value. Surrounding the boxes (shaded area) on each side is a rotated kernel density plot, which is comparable to a histogram with infinitely small bin sizes. The figure was generated using the package ggplot2^41^ in R^42^.

Next, we divided our pTBI patients according to lesion locations into bilateral vmPFC (*n* = 14), dlPFC (*n* = 15), posterior STS/TPJ (*n* = 17) groups and compared their scores on the God Image Inventory to the HCs (N = 31). A one-way ANOVA of the mean God Image Inventory scores showed a significant main effect of group (F(3, 73) = 3.37, p = 0.022, η_p_^2^ = 0.121). Bonferroni post-hoc comparisons demonstrated that the God Image Inventory score was significantly *higher* in the vmPFC group (M = 0.572, SD = 0.492, 95% CI [0.080, 1.064]), compared to the HC group (M = − 0.333, SD = 1.118, 95% CI [− 0.666, − 0.001]), p = 0.029, but not compared to the dlPFC group (M = 0.109, SD = 0.825, 95% CI [− 0.369, 0.558]), p > 0.05), nor the posterior STS/TPJ group (M = 1.165, SD = 0.660, 95% CI [− 0.284, 0.614]), p > 0.05 (Fig. 1). The dlPFC and posterior STS/TPJ groups performed similarly to the HC group.

To further estimate the strength of evidence for our hypotheses, we submitted the God Image score to a Bayesian equivalent of a one-way ANOVA. Then, we compared the Bayes factor (BF_10_) for a group effect relative to a null model (i.e., no effect). Compared to the null model, the model that included the main effect of lesion group in God Image score had a BF_10_ of 2.69 (error % = 0.032) (Table 3). Post-hoc comparisons among groups indicated moderate evidence in favor of a difference between the vmPFC and HC groups on the God Image Score (BF_10,U_ = 4.95, error % = 7.650e−5). See Table 4 for post-hoc results.

**Table 3:** Bayesian one-way ANOVA model comparison for God Image Score.

Models	P(M)	P(M|data)	BF_M_	BF_10_	Error %
Null model	0.500	0.271	0.371	1.000	
Groups	0.500	0.729	2.692	2.692	0.032

**Table 4:** Post Hoc comparisons for god image score.

		Prior odds	Posterior odds	BF_10, U_	Error %
HC	dlPFC	0.414	0.250	0.603	0.009
	pststpj	0.414	0.342	0.826	0.015
	vmPFC	0.414	2.054	4.958	7.650e−5
dlPFC	pststpj	0.414	0.142	0.343	8.165e−5
	vmPFC	0.414	0.459	1.109	0.003
pststpj	vmPFC	0.414	0.488	1.178	0.003

The posterior odds have been corrected for multiple comparisons by fixing to 0.5 the prior probability that the null hypothesis holds across all comparisons^43^. Individual comparisons are based on the default t-test with a Cauchy (0, r = 1/sqrt(2)) prior. The "U" in the Bayes factor denotes that it is uncorrected.

A one-way ANOVA of the mean Interpersonal Reactivity Index scores did not show a significant main effect of group (F(3, 71) = 0.776, p = 0.551).

Next, we investigated how empathy and theory of mind factors could influence the God Image score.

### Correlation analyses

To investigate the relationship between God Image Inventory Score and Empathy Scores (IRIs) we performed the following correlation analyses. Correlations were conducted in all pTBI patients and HCs, and also in lesion subgroups. We performed corresponding Bayesian correlations. Finally, we performed Fischer’s exact test to compare the correlations of pTBIs and HCs.

First, performing lesion-based correlations, God image scores were significantly correlated with the extent of vmPFC (rho = 0.213, p = 0.015) lesion size, but not dlPFC (rho = 0.127, p = 0.187) or pSTS/TPJ (rho = 0.078, p = 0.420) lesion size. Corresponding Bayesian correlations between God image scores and lesion size, showed a BF10 = 1.85 for the vmPFC, BF10 = 0.42 for the dlPFC, and BF10 = 0.23 for the pSTS/TPJ. More extensive lesions to the vmPFC were associated with higher scores on the God image inventory.

Second, performing correlation analyses between empathy subscale and God Image inventory scores, the IRI Empathic Concern scale score was significantly correlated with the God Image Inventory score in both pTBI (rho = 0.22, p = 0.023) and HC (rho = 0.51, p = 0.004) patients. For the other IRI subscales, all showed a p > 0.05 for pTBI and HC patients. A Fischer’s exact test between the correlations of pTBIs and HCs showed a marginally significant difference in the correlation strength between God Image and IRI Empathic Concern (Z = 1.58, p = 0.057), indicating that greater empathy was associated with a more intense connection to divine entities, particularly in HCs. Corresponding Bayesian correlations between God Image scores and IRI subscales in pTBIs showed a positive and strong correlation only with Empathic Concern (BF10 = 47.62), for other subscales all BF10 < 0.6. Corresponding Bayesian correlations between God Image scores and IRI subscales in HCs showed a positive and strong correlation only with Empathic Concern (BF10 = 32.37), for other subscales all BF10 < 0.3. Comparing the God Image-Personal Distress correlations from the vmPFC group and other lesion subgroups using Fischer’s exact test showed that the correlation in the vmPFC group was significantly different from the correlation in the dlPFC group (Z = − 1.75, p = 0.04) and in the pSTS/TPJ group (Z = − 2.536, p = 0.006). After FDR correction for all pairwise correlation tests, the association between God image and the IRI Personal Distress subscale in the vmPFC subgroup was not significant (FDR-adjusted p-value = 0.128).

Investigating the relationship between God image and IRI subscales within the lesion subgroups, the IRI Personal Distress subscale was negatively correlated with the God image score in the vmPFC group (rho = − 0.64, p = 0.018) but not in the other lesion subgroups. Corresponding Bayesian correlations between God Image scores and IRI subscales in vmPFC showed a negative and moderate correlation only with Personal Distress (BF10 = 3.68), for other subscales, the lesion groups had a BF10 < 0.6. This suggests that in patients with vmPFC lesions, a stronger God image score is associated with less agitation, anxiety or distress in the face of an emergency (such as someone else getting hurt or suffering).

Third, investigating the relationship between the God Image Inventory and ToM tests (including the RMET for affective ToM and the Faux Pas Test for cognitive ToM), God image scores did not correlate with cognitive or affective ToM in the pTBI group (FP: rho = − 0.11, p = 0.27; RMET: rho = − 0.16, p = 0.12). However, in HCs, God image scores were negatively correlated with performance on the Faux Pas test (rho = − 0.53, p = 0.0024) but not in the RMET test (rho = − 0.21, p = 0.29). Corresponding Bayesian correlations showed BF10 < 0.4. Corresponding Bayesian correlations showed a BF10 = 0.34 for RMET test and BF10 = 2.02 for the Faux Pas test. These results suggest that better cognitive ToM in HCs was associated with weaker God Image scores (This correlation was performed after removing one outlier > 2.5 standard deviation).

### Mediation analysis

**Figure 2:**
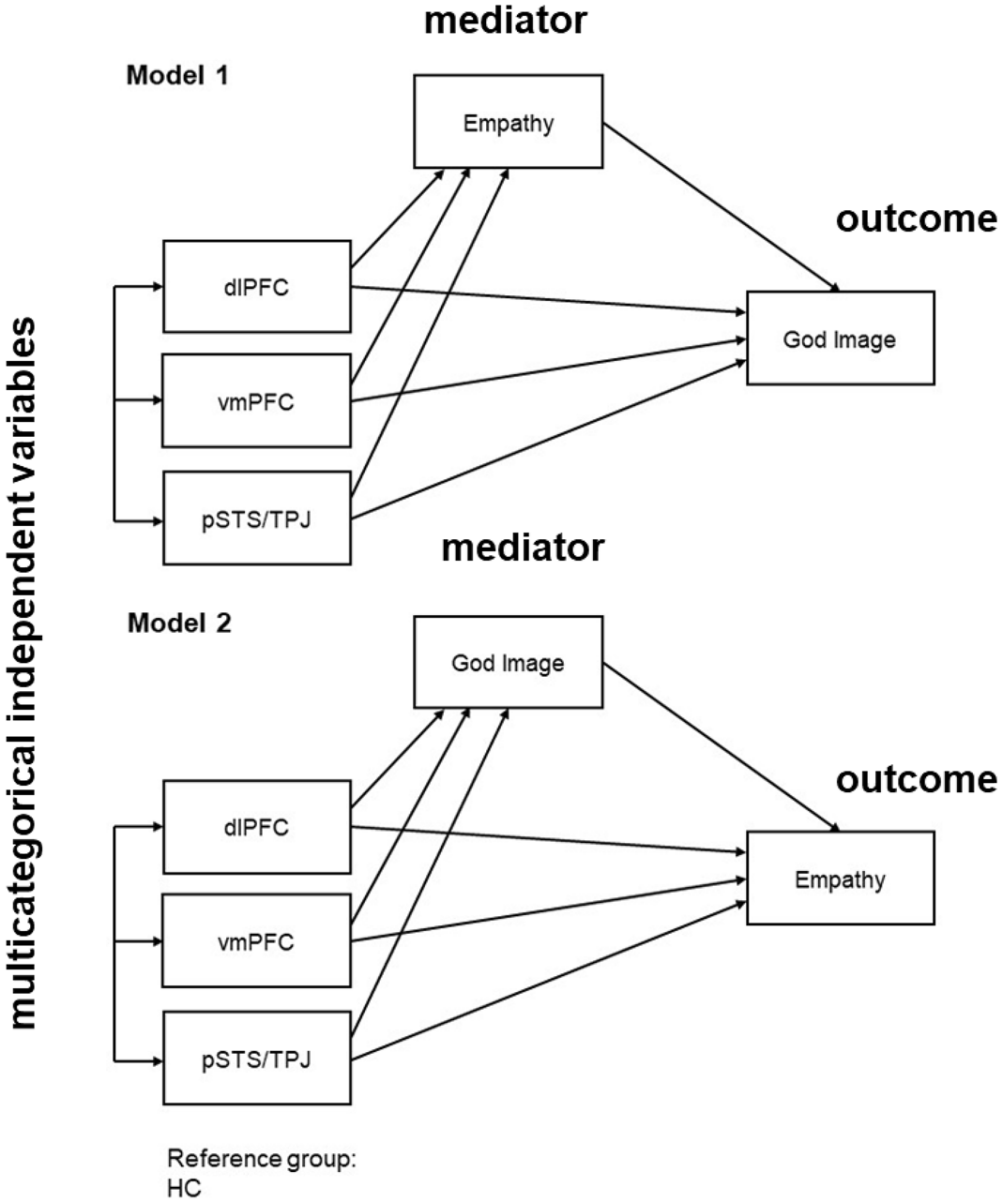
These path diagrams illustrate the two mediation models we used with three multicategorical independent variables, a mediator, and an outcome variable**.**

We applied two mediation models to examine if there was a causal pathway among lesion location, God image and empathy. (Fig. 2, Table 5). Hypothesis 1 predicted that those who believe that God has a close relationship with them are already highly empathetic towards others; therefore, God mirrors their own behavior. Hypothesis 2 predicted that those who belief in God, are more empathetic towards others. The relative indirect effects of Model 1 were not significant, suggesting that there is no significant effect of lesion groups on God image score, mediated through empathy, when compared to the HC group.

**Table 5:** Mediation analyses results showing the indirect effect of lesions in dlPFC, vmPFC and pSTS/TPJ relative to the HCs.

	Indirect effect	SE	95% CI	
**Model 1: X = Groups, M = Empathy, Y = God image (N = 77)**				
dlPFC	− 0.0987	0.1322	− 0.4024	0.1324
vmPFC	0.0562	0.1156	− 0.1569	0.3121
pSTS/TPJ	0.1769	0.1201	− 0.0135	0.4667
**Model 2: X = Groups, M = God image, Y = Empathy (N = 77)**				
dlPFC	0.8918	0.5793	− 0.0037	2.3628
vmPFC	1.7245	0.6524	0.692	3.3301
pSTS/TPJ	0.9915	0.5262	0.1714	2.3093

In contrast, evaluating Model 2, we found a significant relative indirect effect of lesion groups on Empathy mediated through God Image for the vmPFC group and the pSTS/TPJ groups. The results suggest that relative to the HCs, the subjects in the vmPFC group reported higher empathy scores as a result of the positive effect of vmPFC lesions on God image scores, which in turn increased empathy. Similarly, compared to the HCs, the patients in the pSTS/TPJ groups also reported higher empathy scores as a result of the positive effect of an pSTS/TPJ lesion on God image scores which in turn increased empathy.

The results did not change after controlling for post-injury AFQT scores as a covariate. The relative indirect effects of Model 1 were not significant (95% CI dlPFC: [− 0.3791, 0.1769]; vmPFC: [− 0.2826, 0.3458]; pSTS/TPJ: [− 0.0394, 0.4576]). The relative indirect effects for Model 2 were significant for vmPFC and pSTS/TPJ groups (95% CI dlPFC: [− 0.1529, 2.2177]; vmPFC: [0.4557, 3.3435]; pSTS/TPJ: [0.0822, 2.2797]).

We caution that mediation analyses cannot establish with certainty that the causal direction is as proposed between the mediator and outcome. Nevertheless, the absence of indirect effects in Model 1 as opposed to the detected indirect effects in Model 2 does lend more confidence to the latter mediation path. As acknowledged in Hayes^44^, testing alternative causal flows has some utility in discounting at least some competing causal orders of alternative accounts. Even though, our data do not afford causal inference, the patterns we observed are consistent with empathetic components and religious belief affecting social cognition.

## Discussion

The primary goal of this study was to investigate the neural foundation of the relationship between religious belief and empathetic response. To evaluate the hypothesis that religious beliefs support empathic cognition we administered the God image questionnaire, empathy questionnaire, and ToM tasks to individuals with brain damage in major hubs of the ToM network and healthy controls. First, we found that patients with a vmPFC lesion had the highest scores on the God image questionnaire, indicating a stronger sense of belonging with God and the presence of God in their lives. Second, we found that God image scores are correlated with the size of the vmPFC lesion. Third, we discovered a positive association between empathy and God Image inventory scores, consistent with the theory that higher God image scores predicts greater empathy. Fourth, we observed that patients with vmPFC lesions that have higher God image scores also have fewer negative feelings such as agitation, anxiety or distress in the face of an emergency. Fifth, we observed that better cognitive ToM scores were associated with weaker God Image scores. Finally, our findings revealed a statistically significant indirect effect of lesion group on Empathy mediated through the God Image score for both the vmPFC group and the pSTS/TPJ group.

Our observation that individuals with vmPFC lesions have higher scores on items evaluating a sense of God accords with previous research indicating that vmPFC networks compute social evaluation^45–47^ as well as previous research indicating that vmPFC damage increases authoritarianism, religious fundamentalism^45,46^ and aggression^48^. A convergent line of evidence derives from developmental studies, children—where it is well established that their PFC is less developed compared to other brain regions^49^—tend to believe more^50^. On the other hand, older populations with diminished PFC function tend to express higher levels of religious beliefs^51^. In the group analysis, the lack of a statistically reliable association between vmPFC and dlPFC lesion patients limits our inference about the role of these networks in processing social information. Our failure to rule out differences between our subject groups might be because the dlPFC group, which had the fewest members, was underpowered. However, the effects of vmPFC network activations on internalized images of God is corroborated by the positive correlation found between lesion size in the vmPFC (but not dlPFC nor pSTS/TPJ) and the score on the God Image inventory.

Our observation that there is an interplay of religious belief and empathy accords with previous research^52–54^ indicating that God Image inventory scores are positively correlated with empathy (IRI empathic concern). We suggest that higher empathy levels are likely associated with a higher capacity to connect to supernatural agents. Consistent with this inference, we observe that that patients with vmPFC lesions not only have higher God image scores but also reported negative feelings such as agitation, anxiety or distress in the face of an emergency (IRI personal distress). This finding supports theories that religious beliefs function as a coping mechanism to reduce distress and anxiety^45,46^. Our observation that there is a negative correlation between theory of mind (TOM) abilities (Faux pas test) and religiosity in healthy controls contrasts with previous evidence in adolescents that TOM deficits predict lower religious beliefs^40^. This divergence in results may be explained in terms of different age cohorts: the link between God image score and social cognition might only hold in populations where religion is normative, and younger cohorts are less religious than older cohorts^55^.

Our observation that patients in the vmPFC group exhibit a higher indirect effect of god image on empathy compared to the HC groups reveals that for patients with vmPFC lesions a significant amount of the variance in their empathy scores can be attributed to individual differences in their religious beliefs. In our study, patients with vmPFC lesions do not have less empathy (as shown in previous studies using different scales, see Refs.^56,57^). However, when their religious beliefs are considered those with stronger religious beliefs are able to mitigate the negative effect their lesions may have on empathy. This nuanced effect between brain lesion, religion, and empathy reveals the complex neural basis of the relationship between spiritual belief, social integration, and caring for others. Our study suggests that encouraging religious belief, with its role in modulating empathy, could help patients with vmPFC lesions better restore their empathetic tendencies.

Our study cannot conclusively disentangle the role of vmPFC and TPJ. The TPJ effect on religion and empathy is only apparent in the mediation analyses. Most of our statistically significant results revealed the importance of the vmPFC for religiosity and in turn the effect of religiosity on empathy. The vmPFC, known for its role in computing stimulus value^34,58^, could have a central role in scaling the importance of religious tenets that encourage empathy. Further studies, using larger TBI patient samples, or different techniques such as TMS could help in disentangling the specific role of the vmPFC and TPJ in mediating between empathy and religious belief.

Collectively, these findings contribute to social and affective neuroscience by supporting the theory that mental representations of God could amplify empathetic responses. We caution that our findings are limited to a male American sample of war veterans. Further studies merit investigating the relationship between religious belief and empathetic response in other religious and cultural settings. Moreover, our findings should not imply that non-religious people are less empathetic. Empathy may be expressed through different cortical networks than those supporting religious beliefs^16^. Nevertheless, our finding that belief in God may contribute to an empathetic response is consistent with the manifest claims of many religious traditions.

Recent theories of religious prosociality have suggested that punishment typically drives prosocial behavior^59^. Our lesion mapping findings, however, suggest another pathway to prosocial behaviors though empathetic responsivity. Notably an emphasis on empathy is a core feature of religious traditions, both east and west^60^, however this emphasis is explicitly downplayed in cultural evolutionary literatures^61^.

Our mediation analysis findings indicated that in patients with vmPFC lesions, belief in God may enhance empathic responses. This suggests that religious beliefs are not simply the effect of a prosocial orientation, as some have hypothesized^62^. Rather, our observations are consistent with the theory that beliefs in God may promote psychological phenomena such as empathy, a key engine of cooperation among strangers^63^. The vmPFC and TPJ may be especially important neural hubs mediating this influence given their connectivity with dorsal and lateral PFC. When vmPFC and TPJ are damaged, the dlPFC may be freed from the constraints of the person’s routine and integrative behaviors allowing for a spared belief in God to influence empathetic tendencies.

Although lesion-mapping studies are fundamental in determining the causal relationship between brain structure and behavior, our study must be considered in the context of a few limitations. First, we studied male Vietnam War veterans, and hence the results cannot be generalized to the general population (i.e., including females and younger populations). Second, our neuropsychological assessment was performed on Phase IV from 2009 to 2012 (except for TOM measures assessed on Phase III), while our CT scans were performed on Phase III from 2003 to 2006, although a clinical visual inspection of the Phase IV scans by an experienced clinical neurologist participating in the VHIS showed no obvious changes from the Phase III scans in individuals. Third, the causal interpretation is preliminary and more research is needed to fully understand the causal relationships between brain injury, empathy, and religious beliefs.

It is also possible that non-biological factors could explain stronger religious beliefs following brain injury. After a brain injury people might find new interesting activities that could help them cope with a potential loss of occupation, loneliness and isolation. Affiliation to a religious community could help patients suffering from severe brain injury to develop increased religious beliefs. In addition, studies found that spirituality was a predictor for life satisfaction, distress and functional recovery in patients following a traumatic brain injury^64^ and for caregivers’ outcomes^65^.

To conclude, our study suggests that the neural hubs helping to mediate the influence of religious concepts on empathy include regions in the PFC and TPJ. The vmPFC and TPJ, when damaged, likely lead to less influence on the dlPFC and related brain regions, causing the facilitation of religious and social processes subserved by more dorsolateral PFC. This complex neural interplay of several distinct forms of social behavior are fractionated when people have brain damage to the PFC or TPJ leading to modifications of the association between these forms of social behavior.

This finding suggests that religion’s role in driving prosocial responses is not limited to extrinsic fear of supernatural punishments but may also extend to encouraging empathy for other people-at least in their in-group.

More generally, our approach clarifies how the toolkit of social-affective neuroscience may be employed to clarify fundamental questions about the human condition.

## Materials and methods

### Participants

Participants were drawn from phase IV of the W.F. Caveness Vietnam Head Injury Study (VHIS) registry, which is a prospective, long-term follow-up study of male veterans with focal penetrating traumatic brain injury (pTBI) and veterans without injury (healthy control, HC)^66^. There was no intervention in the VHIS and we were never testing a treatment. The VHIS was an observational type study.

**Figure 3:**
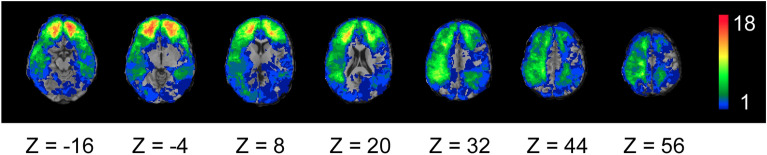
Overlay density map of the pTBI patients’ lesions (n = 109). The color legend indicates number of patients with damage to a particular voxel. Images are in radiological space (i.e., right is left).

**Figure 4:**
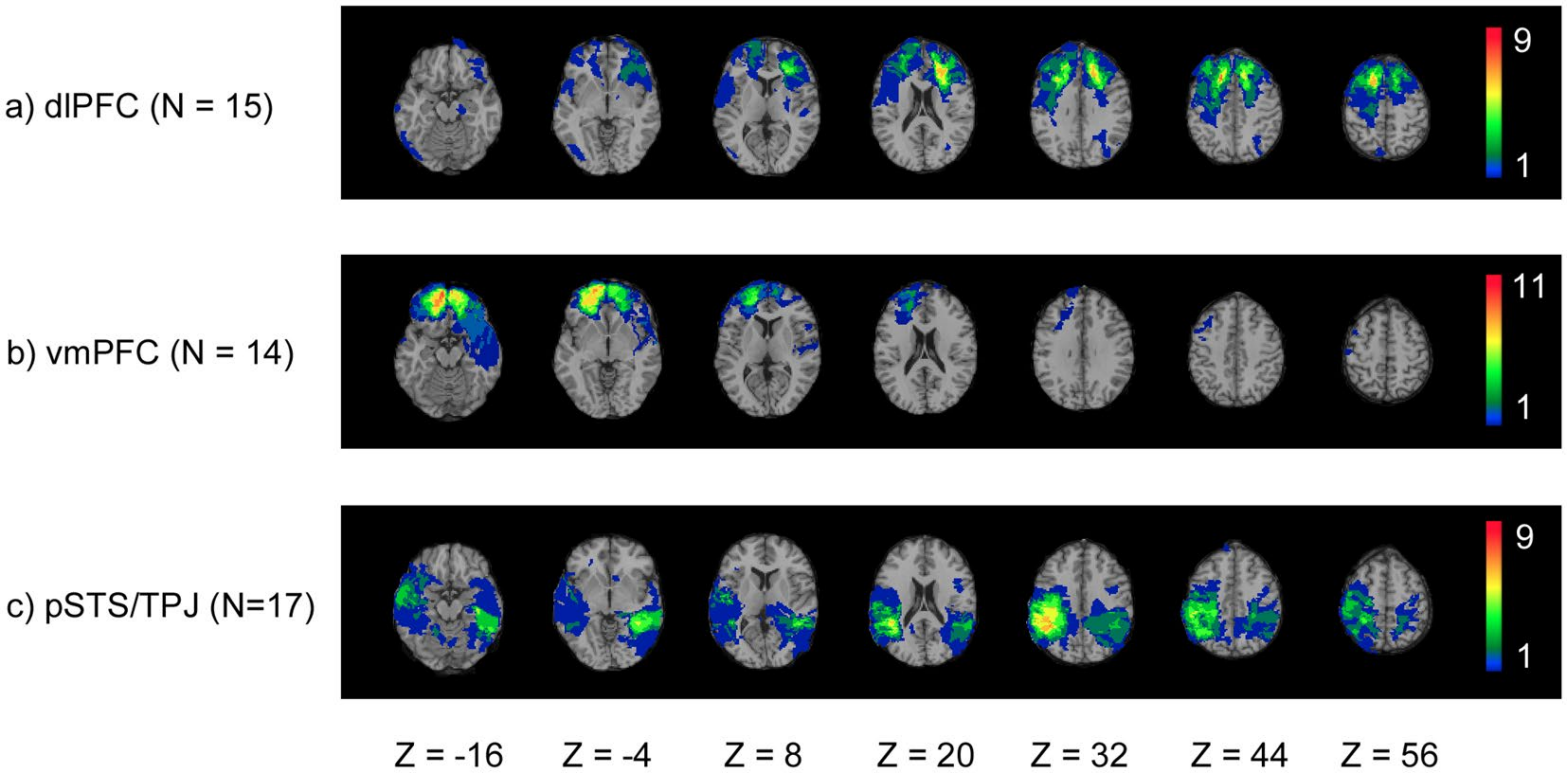
Lesion overlay of three lesion groups with damage to the dlPFC (n = 15), vmPFC (n = 14), or PSTS/TPJ (n = 17). The color legend indicates number of patients with damage to a particular voxel. Images are in radiological space (i.e., right is left).

During phase IV (2008–2012, i.e. approximately 40–45 years post-injury) we assessed 135 pTBI and 35 HC. In total, 109 pTBI patients and *n* = 31 HCs completed the protocols included in this study (religious beliefs and empathy scales).To perform subgroup analyses, we divided our participants into ventromedial prefrontal cortex (vmPFC; *n* = 14), dorsolateral prefrontal cortex (dlPFC; *n* = 15) and posterior superior temporal sulcus/temporaparietal junction (pSTS/TPJ; *n* = 17) lesion subgroups based on the presence or absence of brain damage in these specific areas (Figs. 3 and 4 show overlays for all pTBI patients and lesion groups). The pTBI and HC, as well as the lesion groups and HC were matched with respect to age, level of education, handedness, and pre-injury general intelligence (Tables 1, 2). The study was approved by an Institutional Review Board at the National Institute of Neurological Disorders and Stroke at the National Institute of Health, Bethesda, MD, USA All participants understood the study procedures and provided their written informed consent. All methods were performed in accordance with the relevant guidelines and regulations.

### CT acquisition and analysis

Axial CT scans without contrast were acquired on a GE Medical Systems Light Speed Plus CT scanner in helical mode at the Bethesda Naval Hospital, Bethesda, MD. All scans were performed during Phase III of the Vietnam Head Injury Study [VHIS; (2003–2006)]. Structural neuroimaging data were reconstructed with an in-plane voxel size of 0.4 × 0.4 mm, an overlapping slice thickness of 2.5 mm, and a 1-mm slice interval. Lesion location and volume from CT images were determined using the interactive ABLe software^67^, implemented in MEDx v3.44 (Medical Numerics) with enhancements to support the Automated Anatomical Labeling (AAL) atlas^68^.

The CT image of each subject’s brain was normalized to a CT template brain image in Montreal Neurological Institute (MNI) space. Lesion volume was calculated by manually tracing the lesion in all relevant slices of the CT image in native space and then summing the trace areas and multiplying by slice thickness. Manual tracing was performed by a trained psychiatrist with clinical experience of reading CT scans. It was then reviewed by an observer that was blind to the results of the clinical evaluation and neuropsychological testing (J.G.), enabling a consensus decision to be reached regarding the limits of each lesion.

We quantified the percentage of AAL structures impacted by the lesion from the overlap of the normalized lesion images with the AAL atlas. In addition, we defined regions of interest (ROIs) in the vmPFC, dlPFC, and pSTS/TPJ as previously described [Ref.^69^ for vmPFC and dlPFC^70^ for pSTS/TPJ], by identifying AAL structures within specified ranges of MNI coordinates. See Fig. 4 for group lesion overlays.

### Neuropsychological testing

Participants were assessed from 2009 to 2012 at the National Institutes of Health in Bethesda, MD, over a 5- to 7-day period with tests that measured a wide variety of neuropsychological functions including memory, language, executive functioning, and social cognition. For this study, we focused on the assessment of religious beliefs and empathy.

As part of their one-week Phase 4 evaluation, participants completed 14 items from the God Image Inventory^37^. This inventory reflects an internal model of the sort of person that the individual imagines God to be. The original God Image Inventory consists of 156 items^37^. Since our participants were assessed in a variety of tasks and questionnaires over a week, we decided to select items from the Presence and Salience subscales. The Cronbach’s alpha for the God Image Inventory is 0.958. The participants rated the items on a 4-point Likert scale, ranging from 1 = “Strongly disagree” to 4 = “Strongly agree”. Examples of the items are: “*I can talk to God on an intimate basis”*, “*God tells me what he wants from me”*. Most of the items were related to the personal and intimate relationship between God and the individual (i.e., Presence dimension). A higher score indicates a stronger sense of belonging with God and the presence of God. See the Appendix for the complete list of God Image Inventory items.

To assess empathy, we used the Interpersonal Reactivity Index (IRI) that defines empathy as the “reactions of one individual to the observed experiences of another”^71^. The IRI consists of 28-items answered on a 5-point Likert scale ranging from “Does not describe me well” to “Describes me very well”. The measure has 4 subscales, each made up of 7 different items: Perspective Taking—the tendency to spontaneously adopt the psychological point of view of others; Fantasy—the tendency to transpose oneself imaginatively into the feelings and actions of fictitious characters in books, movies, and plays; Empathic Concern—"other-oriented" feelings of sympathy and concern for unfortunate others; Personal Distress—"self-oriented" feelings of personal anxiety and unease in tense interpersonal settings. This measure has been previously used to identify empathic impairments in participants with frontotemporal dementia^72^, and traumatic brain injury^73^; and has been demonstrated to have good test–retest reliability and internal reliability^71^. Cronbach’s alpha for the IRI subscales generally ranges between 0.70 to 0.78^74^.

To assess Theory of Mind (ToM), we used the Reading the Mind in the Eyes Test [RMET^75^] for affective ToM (from Phase IV), and the Faux Pas Recognition Task [FPRT^76^] Test for cognitive ToM (from Phase III). The RMET is a recognized measure of adult mentalizing, consisting of 36 black-and-white photographs (18 males and 18 females) of the eye region of faces, each presented along with four adjective choices, one of which describes the mental state expressed in each image. Stimuli were presented using the software package E-Prime (Psychology Software Tools Sharpsburg, PA, USA, https://www.pstnet.com/). In each of the 36 trials, participants saw both a photo showing only the eye region of face and four adjectives. Participants were asked to choose the adjective that best described the mental states inferred by the eyes (i.e., accusing, aghast, amused, apologetic, arrogant, bewildered, cautious, confused, decisive, eager, embarrassed, guilty, and horrified).

The FPRT is a subtle measure of social reasoning, consisting of 20 short (one paragraph) stories: 10 stories with a Faux Pas (the speaker unintentionally said something hurtful or insulting to the listener) and 10 stories without a Faux Pas. Only the 10 Faux Pas stories were evaluated in this study^77,78^. For each trial, the experimenter read the story to the participants, while they read along on their own copy. To reduce memory demands, the stories remained in front of the participant during the duration of the task. After each story, participants were asked a series of questions.

#### Control measures

We evaluated pre- and post-injury general intelligence using the Armed Forces Qualification Test [AFQT-7A^79^], verbal fluency using the Verbal Fluency Delis–Kaplan Executive Function System [DKEFS^80^], verbal comprehension using the Token Test [TT^81^], post-traumatic stress using the Mississippi Post-Traumatic Stress Disorder [PTSD^82^], and depression using the Beck Depression Inventory-II [BDI-II^83^].

### Statistical analyses

Behavioral data analysis was carried out using SPSS 11.0 (www.spss.com; SPSS), with the alpha level set to P < 0.05 (one-tailed). Data were tested for Gaussian distribution (Kolmogorov–Smirnov test) and variance homogeneity (Bartlett’s test). Unless otherwise specified, data were normally distributed, and assumptions for analyses of variance were not violated.

Two-sample independent t-tests were performed to compare the performance on the God Image Inventory (religious belief) and Interpersonal Reactivity Index (Empathy) between pTBI and HC. One-way ANOVAs were performed to compare God Image Inventory and Interpersonal Reactivity Index scores among the different lesion subgroups and HC, with Bonferroni adjustment applied to post-hoc pairwise comparisons.

We complemented this frequentist statistical analysis approach by also examining the main results using a Bayesian approach. As opposed to classical null hypothesis significance testing, Bayesian analyses: (1) can quantify evidence in favor of H0 (null hypothesis), (2) allows comparison between different models [e.g., H0 vs H1 (alternative hypothesis)], and (3) is not biased against H0, unlike classical null hypothesis significance testing^84–86^. We used a Bayesian independent sample t-test, an equivalent to a classical two sample independent t-test^84^, and a one-way ANOVA, which is an equivalent to a classical one-way ANOVA^85^. We used God Image Factor Score as the dependent variable, and pTBI vs HC as groups for the Bayesian independent sample t-test, and dlPFC, vmPFC, STS/TPJ, and HC as groups for the Bayesian one-way ANOVA. Bayesian statistical analyses were conducted in the JASP (2019) software package v 0.9.2.0^87^.

For both the Bayesian ANOVA and the t tests, we used the default priors (as implemented in JASP) as they place data points in realistic ranges without being overcommitted to any one point. In addition, they fit a large set of psychological data with moderate effect sizes, and carry a minimum degree of information^84,85^. The Bayes factor (BF) represents an odds ratio, i.e., the probability of the data under one hypothesis relative to another. As example, a value of BF_10_ = 6 designates the data are six times more likely under H1 than H0. While, a value of BF_01_ = 3 designates the data are three times more likely under H0 than H1. Our interpretation of BF values follows the standard recommendations^88,89^, i.e., a BF value ranging from 1 to 3 infers moderate evidence, from 3 to 10 substantial evidence, and from 10 to 30 strong evidence. BF_10_ calculates evidence for the alternative hypothesis (H1) relative to the null hypothesis (H0), while BF_01_ calculates evidence for the null hypothesis (H0) relative to the alternative hypothesis (H1).

Correlation analyses were performed using Spearman’s rho correlations to assess the relationship between God image scores and empathy, ToM, as well as lesion volume percentage. Correlations between God image scores, empathy and ToM were conducted in all pTBI patients and HCs, and also in lesion subgroups. We performed corresponding Bayesian correlations. Finally, we performed Fischer’s exact test to compare the correlations of pTBIs and HCs.

Mediation analyses were performed to test whether there was a causal path between location of brain lesion, God image and empathy. We ran two mediation models to test two alternative hypotheses. Hypothesis 1 predicts that those who believe that God has a close relationship with them are already highly empathetic towards others; therefore, God mirrors their own behavior. Hypothesis 2 predicts that those who belief in God, are more empathetic towards others. In recent recommendations and practices for mediation analyses, the assumption for an overall relationship between predictor and outcome is no longer considered necessary^64–66^. The only requirement needed to establish a mediation effect is a significant indirect effect, which we specifically hypothesized and went on to assess with bootstrapping.

We used the PROCESS macro implemented in SPSS (Hayes & Preacher, 2014)^90^ to conduct mediation analyses, following the procedure described in Hayes & Preacher 2014. We specified mediation models with lesion and HC subgroup identity as the multicategorical independent variable, which indicated whether a participant belonged to the dlPFC, vmPFC, pSTS/TPJ lesion or HC group. We used the dummy coding scheme with HC as the reference level. Thus, the relative indirect effects are the indirect effect of the selected lesion group when compared to the HC group.

To test Hypothesis 1, we entered empathy as the mediator and God image score as the dependent variable. Alternatively, to test Hypothesis 2, we entered God image as the mediator and empathy as the dependent variable. We used 5000 bootstrap samples to evaluate the bias-corrected 95% confidence intervals of the size of the relative indirect effects.

## Data Availability

The data that support the findings of this study are available on request from the corresponding author, [IC, or JG]. The data are not publicly available due to privacy restrictions.

## Appendix

### List of God Image Inventory items (* reversed score items)

- *God does not notice me.
- God lifts me up.
- *I am never really sure that God is really listening to me.
- *God doesn’t feel very personal to me.
- I can talk to God on an intimate basis.
- *I get no feeling of closeness to God, even in prayer.
- I feel that God knows me by name.
- *God never reached out to me.
- I feel warm inside when I pray.
- *God does not answer when I call.
- Prayer is very meaningful to me.
- *I prefer to face my problems without prayer.
- God tells me what he wants from me.
- *I do not think about God very often.
